# Taking a fresh look at FAIR for research software

**DOI:** 10.1016/j.patter.2021.100222

**Published:** 2021-03-12

**Authors:** Daniel S. Katz, Morane Gruenpeter, Tom Honeyman

**Affiliations:** 1 University of Illinois, Urbana, IL, USA; 2 Inria, Software Heritage, Paris, France; 3 Australian Research Data Commons, Melbourne, VIC 3145, Australia

## Abstract

Software is increasingly essential in most research, and much of this software is developed specifically for and during research. To make this research software findable, accessible, interoperable, and reusable (FAIR), we need to define exactly what FAIR means for research software and acknowledge that software is a living and complex object for which it is impossible to propose one solution that fits all software.

## Main text

Software is increasingly essential in most research, and much of this software is developed specifically for and during research. If we imagine a world where all research is reproducible, all research software is usable by others (for their own research), all contributors to research software are recognized for their work, all research software is sustained as long as it is useful, and all research software is high quality and robust, one step in this direction is to make research software findable, accessible, interoperable, and reusable (FAIR), which could be done by riding the coattails of the both publicly and governmentally pushed FAIR movement. But to achieve this, we need to define exactly what FAIR means for research software and acknowledge that software is a living and complex object for which it is impossible to propose one solution that fits all software.

In 2016, Wilkinson et al. published a set of principles that defined FAIR for research data.^1^ However, while software can be stored as data, it is not just data.^2^ For example, software is executable, while data are not; software provides a tool, while data provide evidence; software is usually classed as a creative work, while the status of data in copyright law is unclear in many jurisdictions, which leads to software and data requiring the application of different licenses; and software is developed, maintained, and published in different ways than data, often in the open on development platforms that encourage sharing and collaboration, while data are often shared through read-only repositories, leading to differences in versioning, authorships, archiving, reviewing, and publishing.

Under the auspices of FORCE11, the Research Data Alliance (RDA), and the Research Software Alliance (ReSA), a FAIR for Research Software (FAIR4RS) working group (https://www.rd-alliance.org/groups/fair-4-research-software-fair4rs-wg) formed to develop a set of FAIR principles for research software, with a desired follow-on goal of pushing the principles into implementation. In its initial work, the group formed four subgroups to examine different aspects of FAIR for research software that will be combined into a set of principles. Given some prior work on this subject by Lamprecht et al.,^3^ one subgroup examined how this work has been used and is being interpreted, while other subgroups worked to define research software itself and to understand how the FAIR principles are being applied to other types of digital objects, and one subgroup took a fresh look at the problem, initially putting aside Lamprecht et al.’s work and simply starting with the original FAIR data principles. This article discusses that subgroup’s work.^4^

Overall, the group worked by having each member initially vote on if each the four foundational principles and 15 guiding principles applied to research software as written, applied but needed changes, or didn’t apply. After these results were compiled, the group members used a shared document to discuss their reasoning for each. This was then summarized as an initial set of FAIR principles for research software (Table 1), with iterations for the full subgroup to comment, a smaller set of participants to meet to work through differences, and then a final period of the full subgroup commenting, leading to this suggested set of principles.

**Table 1:** FAIR principles and FAIR research software principles

FAIR principles^1^ as listed by GO FAIR	FAIR research software principles,^4^ changes are underlined
**F. Findable**	**F. Findable**

The first step in (re)using data is to find them. Metadata and data should be easy to find for both humans and computers. Machine-readable metadata are essential for automatic discovery of datasets and services, so this is an essential component of the FAIRification process.	The first step in (re)using software is to find it. Metadata and software should be easy to find for both humans and computers. Machine-readable metadata are essential for automatic discovery of software, so this is an essential component of the FAIRification process.
F1. (Meta)data are assigned a globally unique and persistent identifier	F1. Software is assigned a globally unique and persistent identifier
F2. Data are described with rich metadata (defined by R1 below)	F2. Software is described with rich metadata (defined first by R1 below, and then by the original FAIR principles for metadata)
F3. Metadata clearly and explicitly include the identifier of the data they describe	F3. Metadata clearly and explicitly include the identifier of the software they describe
F4. (Meta)data are registered or indexed in a searchable resource	F4. Software is registered or indexed in a searchable resource

**A. Accessible**	**A. Accessible**

Once the user finds the required data, she/he needs to know how can they be accessed, possibly including authentication and authorisation.	Once the user finds the required software, they need to know how it can be accessed, possibly including authentication and authorization.
A1. (Meta)data are retrievable by their identifier using a standardized communications protocol	A1. Software is retrievable by its identifier using a standardized communications protocol
A1.1. The protocol is open, free, and universally implementable	A1.1. The protocol is open, free, and universally implementable
A1.2. The protocol allows for an authentication and authorization procedure, where necessary	A1.2. The protocol allows for an authentication and authorization procedure, where necessary
A2. Metadata are accessible, even when the data are no longer available	A2. Metadata are accessible, even when the software is no longer available

**I. Interoperable**	**I. Interoperable**

The data usually need to be integrated with other data. In addition, the data need to interoperate with applications or workflows for analysis, storage, and processing.	The software usually needs to communicate with other software via exchanged data (or possibly its metadata). Software tools can interoperate via common support for the data they exchange.
I1. (Meta)data use a formal, accessible, shared, and broadly applicable language for knowledge representation.	(deemed unnecessary)
I2. (Meta)data use vocabularies that follow FAIR principles	(deemed unnecessary)
*R1.3. [(Meta)data meet domain-relevant community standards] used as a model for a new Interoperability guiding principle*	I1. Software should read, write or exchange data in a way that meets domain-relevant community standards
I3. (Meta)data include qualified references to other (meta)data	I2. Software includes qualified references to other objects.

**R. Reusable**	**R. Reusable**

The ultimate goal of FAIR is to optimize the reuse of data. To achieve this, metadata and data should be well-described so that they can be replicated and/or combined in different settings.	The ultimate goal of FAIR is to enable and encourage the use and reuse of software. To achieve this, software should be well-described (by metadata) and appropriately structured so that it can be replicated, combined, reinterpreted, reimplemented, and/or used in different settings.
R1. (Meta)data are richly described with a plurality of accurate and relevant attributes	R1. Software is richly described with a plurality of accurate and relevant attributes
R1.1. (Meta)data are released with a clear and accessible data usage license	R1.1. Software is made available with a clear and accessible software usage license
R1.2. (Meta)data are associated with detailed provenance	R1.2. Software is associated with detailed provenance
R1.3. (Meta)data meet domain-relevant community standards	R1.3. Software meets domain-relevant community standards
*I3. [(Meta)data include qualified references to other (meta)data] used as a model for a new Reusability guiding principle*	R2. Software includes qualified references to other software

Overall, we found that many of the principles remained relatively intact as written, as long as considerable interpretation was provided. This was particularly the case for the findable and accessible foundational principles. We found that interoperability and reusability are particularly prone to a broad, overlapping, and sometimes opposing sets of interpretations as written. We have differentiated the two, limiting interoperability to be concerned with the capacity to exchange data between independent software and reusability (implicitly including usability) to be concerned with the relationship between a piece of software and the external software upon which it depends in order to operate (i.e., its dependencies). We propose two new principles modeled on existing ones and provide modified guiding text for these principles to help clarify our final interpretation.

A series of systemic gaps were captured during this process, which include both gaps in understanding and agreement and gaps in systems. Many of these could be considered challenges to implementation of FAIR for research software as much as challenges in defining the principles themselves, as these concepts are interlinked: the principles define what is possible in the context of implementation, while the implementation depends on how the principles are defined. These gaps include identifiers and metadata for software, metadata and identifier authority, identification targets, software structure complexity, documentation, and binaries versus source code.

**Figure 1:**
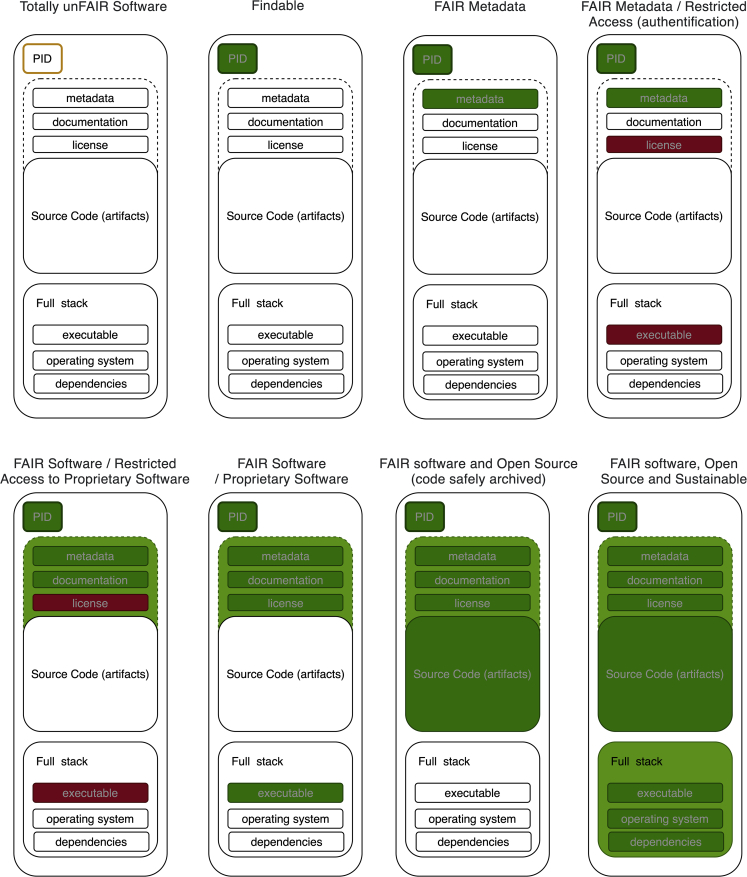
Summarizing software as increasingly FAIR research objects Inspired by the FORCE11 diagram.^5^

Finally, the FAIR principles for research software are a step forward on the path to recognizing software outputs in academia and improving the curation workflows to produce better outputs. Yet FAIR software can’t guarantee executability, robustness, and computational reproducibility, which are goals we want to achieve; doing so requires more than just the FAIR software principles. Figure 1 shows how software is a complex living object composed of different elements and that this can help us use both existing software norms and the FAIR principles to move through FAIR research software to reproducible research.

The next steps for the overall working group are to combine the work of this subgroup with the work of the other subgroups, which will naturally include comparing with Lamprecht et al.’s work and understanding the sources of differences, along with defining research software. We will also consider other 2020 reports, such as the FAIRsFAIR report^6^ and the EOSC Scholarly Infrastructures of Research Software report.^7^ This is intended to lead to a consensus set of FAIR principles for research software, of which this set is one initial step.

## Web resources

FAIR for Research Software working group, https://www.rd-alliance.org/groups/fair-4-research-software-fair4rs-wg
