# Taking a fresh look at FAIR for research software

**DOI:** 10.1016/j.patter.2021.100267

**Published:** 2021-05-14

**Authors:** Daniel S. Katz, Morane Gruenpeter, Tom Honeyman

## Main text

(Patterns *2*, 100222-1–100222-4; March 12, 2021)

In the preparation of this opinion, a draft of Figure 1 was submitted instead of the final version. Figure 1 has now been corrected online and appears below. This change does not affect the conclusions of the paper. The authors sincerely apologize to the readers for any confusion that may have resulted from this error.

**Figure:**
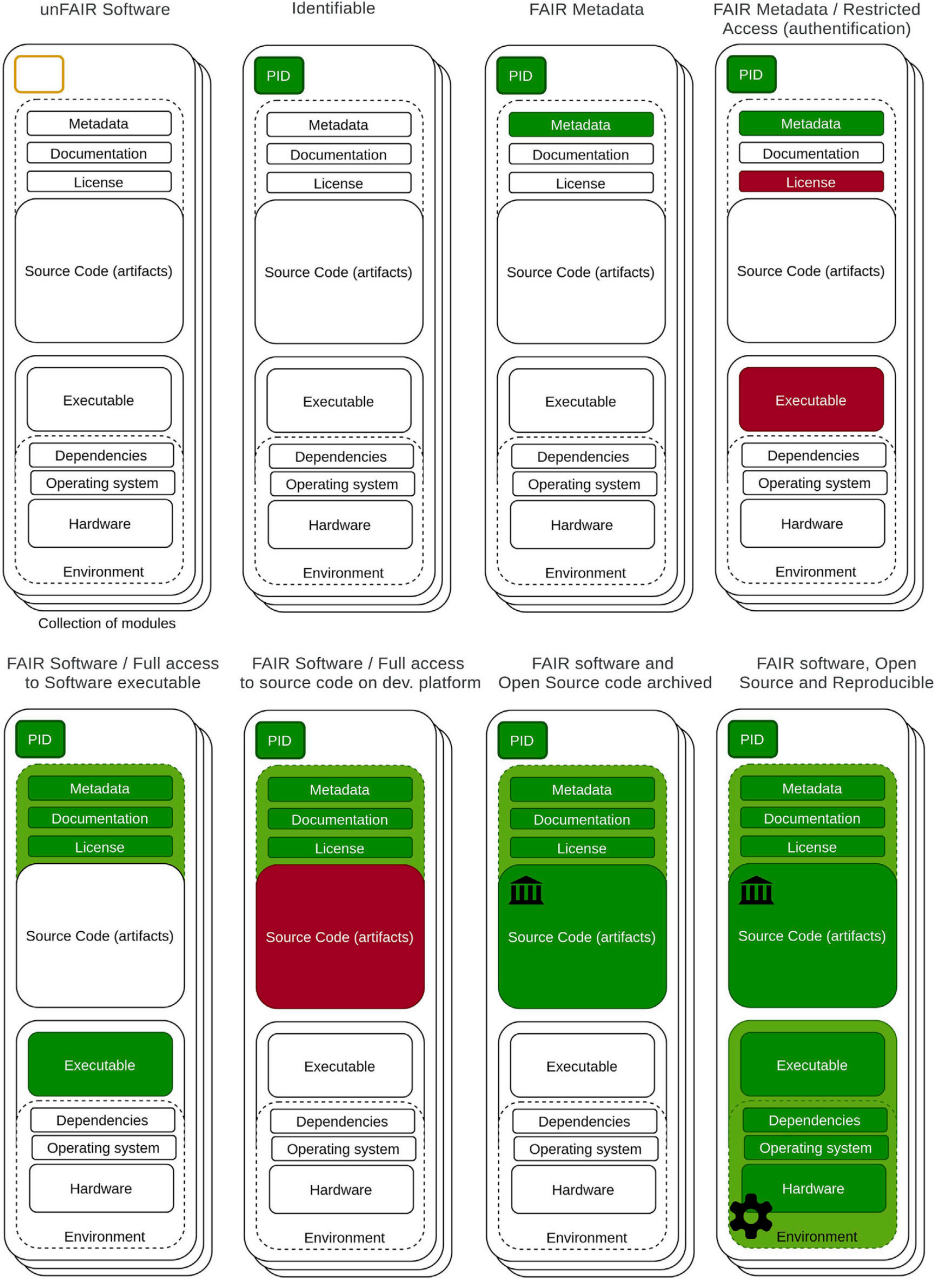
Figure 1. Summarizing software as increasingly FAIR research objects (corrected)

**Figure:**
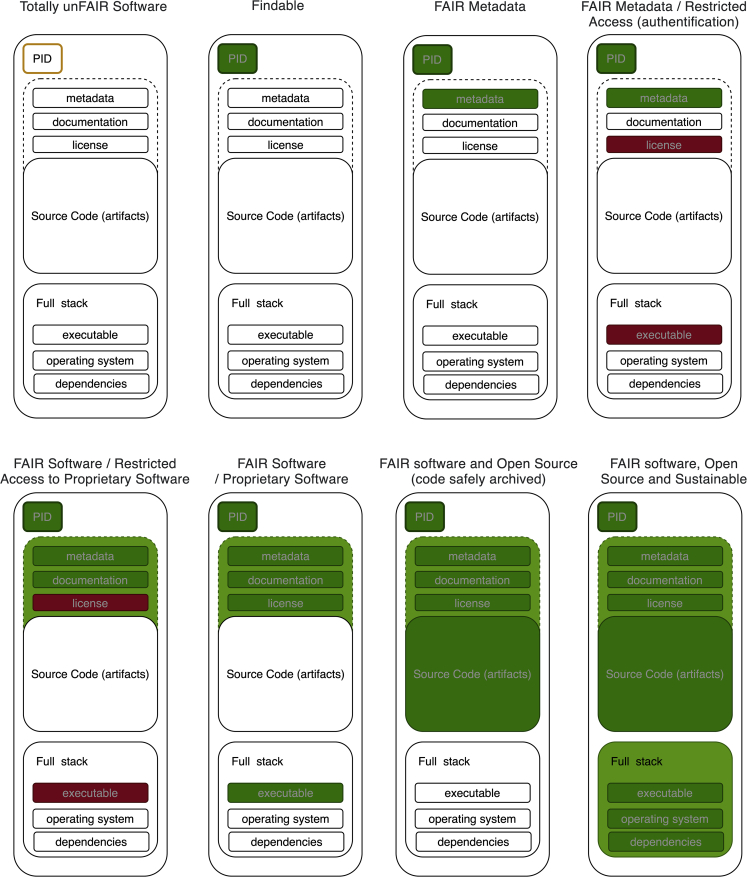
Figure 1. Summarizing software as increasingly FAIR research objects (original)

